# Chemoradiation versus oesophagectomy for locally advanced oesophageal cancer in Chinese patients: study protocol for a randomised controlled trial

**DOI:** 10.1186/s13063-019-3316-5

**Published:** 2019-04-11

**Authors:** Ruinuo Jia, Weijiao Yin, Shuoguo Li, Ruonan Li, Junqiang Yang, Tanyou Shan, Dan Zhou, Wei Wang, Lixin Wan, Fuyou Zhou, Shegan Gao

**Affiliations:** 1 The First Affiliated Hospital, and College of Clinical Medicine of Henan University of Science and Technology, 24 Jinghua Road, Luoyang, 471003 China; 2 Biotherapy Centre and Cancer Centre, The First Affiliated Hospital of Zhengzhou University, China Cancer Hospital, Zhengzhou, China; 3 Nanyang Central Hospital, Nanyang, China; 4 Anyang Tumour Hospital, The Affiliated Hospital of Henan University of Science and Technology, Anyang, China

**Keywords:** Oesophageal squamous cell carcinoma, Definitive chemoradiotherapy, Oesophagectomy

## Abstract

**Background:**

Surgery is the gold standard treatment for local advanced disease, while definitive concurrent chemoradiotherapy (DCRT) is recommended for those who are medically unable to tolerate major surgery or medically fit patients who decline surgery. The primary aim of this trial is to compare the outcomes in Chinese patients with oesophageal squamous cell cancer with locally advanced resectable disease who have received either surgery or DCRT.

**Methods/design:**

One hundred ninety-six patients with T1bN + M0 or T2-4aN0-2 M0 oesophageal squamous cell cancer will be randomised to the DCRT group or the surgery group. In the DCRT group, patients will be given intensity-modulated radiation therapy (IMRT) with 50 Gy/25 fractions and basic chemotherapy with 5-fluorouracil regimens. In the surgery group, patients will receive neoadjuvant chemoradiotherapy (NCRT) and standard oesophagectomy. Five years of follow-up will be scheduled for patients. The primary endpoints are 2-year/5-year overall survival; the secondary endpoints are 2-year/5-year progression-free survival, treatment-related adverse events and the patients’ quality of life. The main evaluation methods include oesophagoscopy, endoscopic ultrasonography and biopsy, oesophageal barium meal, computed tomography, positron emission tomography-computed tomography, blood tests and questionnaires.

**Discussion:**

The preponderant oesophageal cancer pathology type is dramatically different in western Caucasian and Asian oesophageal cancer patients: Caucasian patients present with 80% adenocarcinomas, and Asians patients present with 95% squamous cell carcinomas. This phenomenon needs more in-depth studies to elucidate the differences in these populations. Based on the results of this study, we will show whether DCRT will benefit patients more than oesophagectomy. This study will contribute more evidence to the management of oesophageal squamous cell cancer.

**Trial registration:**

ClinicalTrials.gov, NCT02972372. Registered on 26 November 2016.

**Electronic supplementary material:**

The online version of this article (10.1186/s13063-019-3316-5) contains supplementary material, which is available to authorized users.

## Background

Oesophageal cancer (EC) is the eighth most common cancer worldwide and the sixth leading cause of cancer-related death [1]. In 2012, 456,000 EC cases occurred, and 400,000 people died from EC worldwide. It’s remarkable that almost half of them came from China, with 223,000 EC cases and 197,000 death cases in 2012. Both the highest incidence and highest mortality, 379/100,000 and 150/100,000, respectively, were in Henan, China (including Linzhou, Anyang and Huixian cities). Additionally, EC is the fourth most deadly cancer among men in China; it is responsible for 9.8% of all cancer deaths annually [2].

Worldwide, approximately half of these patients present with locally advanced disease [3]. Radical oesophagectomy remains the most popular treatment for this disease, but the long-term survival is still barely satisfactory [4, 5]. The mortality in the perioperative period is approximately 5% at renowned centres. The 5-year survival rate in patients with oesophageal carcinoma treated by surgery alone is just 10–20% [6]. Numerous clinical studies in past decades have used adjuvant or neoadjuvant chemotherapy and radiotherapy as a tool to improve the clinical outcome of surgery [7]. However, the results from prospective randomised trials on neoadjuvant radiotherapy or chemotherapy alone were not satisfactory [8]. There was no survival benefit associated with these approaches [9].

Concurrent chemoradiotherapy (CRT) as a neoadjuvant therapy has preferable clinical efficacy and has become the standard treatment for local advanced EC with recognised guidelines [10–12]. Compared with chemotherapy or radiation alone, CRT has outstanding advantages. Not only does it achieve a higher rate of complete pathologic regression of oesophageal tumours, but it is also associated with a significant survival benefit [12]. A complete tumour response was frequently observed after neoadjuvant CRT (NCRT), and this has prompted investigations on the role of definitive concurrent chemoradiotherapy (DCRT) in locally advanced oesophageal carcinoma. However, most of those studies came from western, developed countries, and the majority of patients had adenocarcinomas. Different cell pathologies of the tumours could influence the clinical outcome following the same treatment strategy. In recent studies, neoadjuvant or adjuvant chemotherapy was associated with survival benefits in patients suffering from adenocarcinomas of the oesophagus. A prospective clinical study from Hong Kong University investigated the efficacy of CRT (three-dimensional conformal radiation therapy (3DCRT) in combination with two cycles of 5-fluorouracil (5-FU) and cisplatin) compared with surgery in oesophageal squamous cell cancer. Though this trial had shown no significant difference in the 2-year overall survival (OS) between the two study arms, a superior 5-year OS was found in the CRT arm, but with no statistical significance [13, 14].

In recent studies, both oxaliplatin and capecitabine have been shown to be at least equivalent to cisplatin and 5-FU in the treatment of advanced upper gastrointestinal (GI) cancer or EC. Also, they can be given as a convenient 2-h infusion and an oral administration, and they have a more favourable toxicity profile compared to cisplatin and 5-FU [15, 16]. Based on these previous findings, this randomised, open-label, multicentre clinical trial aims to compare outcomes in Chinese patients with locally advanced resectable oesophageal squamous cell cancer (ESCC) who have received either NCRT plus surgery or DCRT. The primary endpoints will be 2-year/5-year OS, and the secondary endpoints will be 2-year/5-year progression-free survival (PFS), treatment-related adverse events (AEs) and the patients’ quality of life (QoL). Additionally, in the subgroup analysis of CRT, we will investigate the effect and AEs between the different chemotherapy regimens: Xelox (capecitabine + oxaliplatin), PF (cisplatin + 5-FU) and single capecitabine. This is the first head-to-head clinical trial to compare CRT with radical operation in Chinese mainland people with locally advanced ESCC.

## Methods/design

### Study design

**Fig. 1:**
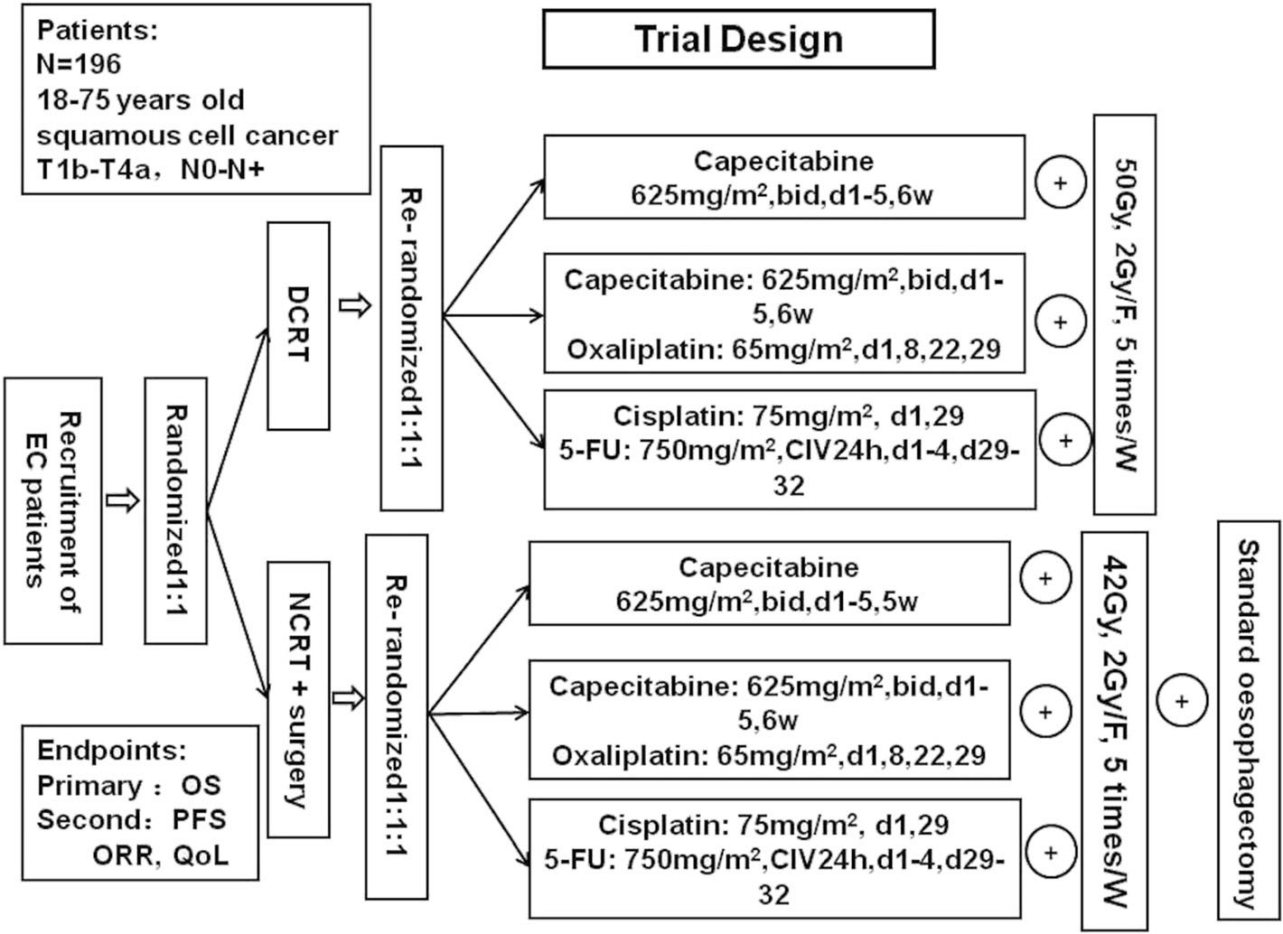
Flow chart of the trial

A multicentre, open, prospective, randomised controlled trial will be conducted that includes three regional hospitals in Henan, which is the area with the highest incidence of ESCC in the world, including the First Affiliated Hospital of Henan University of Science and Technology (HUST), Anyang Tumour Hospital of HUST and Nanyang Centre Hospital. A total population of 216 × 10^5^ is served by these three hospitals. The trial flow chart is shown in Fig. 1. The Standard Protocol Items: Recommendations for Interventional Trials (SPIRIT) checklist is provided in Additional file 1.

### Ethical approval

This study protocol was approved by the Ethics Committees of the First Affiliated Hospital of HUST, Anyang Tumour Hospital of HUST and Nanyang Centre Hospital. The study is performed in accordance with the Declaration of Helsinki, and written informed consent will be collected from each study participant prior to enrolment.

### Study participants

#### Setting

The study will take place at the First Affiliated Hospital of HUST, Anyang Tumour Hospital of HUST and Nanyang Centre Hospital.

#### Patients

A total of 196 ESCC patients with T1bN + M0 or T2-4aN0-2 M0 will be randomised to the CRT group or the surgery group.

#### Inclusion criteria

The inclusion criteria are as follows:

1. Age 18–75 years
2. Mainland Chinese
3. Oesophageal squamous cell cancer confirmed by histology
4. Tumour is resectable
5. Clinical stage cT1bN + M0 or cT2-4aN0-2 M0
6. Performance status score 0–2.

#### Exclusion criteria

The exclusion criteria include the following:

1. Patient has distant metastasis to solid visceral organs or local invasion into the trachea, descending aorta or recurrent laryngeal nerve
2. Patient has a serious premorbid condition or a poor physical status that compromises the thoracotomy
3. Compromised cardiac function or creatinine clearance less than 50 ml/min
4. Maximal voluntary ventilation (MVV) of pulmonary function test is less than 30%.

#### Withdrawal criteria

Patients will be withdrawn from the study if they withdraw informed consent and decline to continue treatment or follow-up.

#### Recruitment

Recruitment will be from cancer centres of the First Affiliated Hospital of HUST, Anyang Tumour Hospital of HUST and Nanyang Centre Hospital, Henan province, China. Research staff will regularly check the inpatient - registry information system and identify any potentially eligible patients. They will liaise with an oncologist to ensure that the patient’s history and screening results are clear for study commencement. Eligible participants who present at the cancer centre when research staff are present will complete informed consent documentation after discussion with the oncologist, fill out baseline measures and then be randomly allocated to the DCRT group or the NCRT plus surgery group.

#### Randomisation

The randomisation codes will be generated by the study statistician using computer-generated random numbers. Participants will be randomly allocated to the DCRT or the NCRT plus surgery group in 1:1 order. Then in each group, participants will be secondarily randomly allocated to subgroups with one of three different chemotherapy regimens in 1:1:1 order.

#### Pretreatment investigations

Patients will receive further staging workup, including oesophagoscopy, endoscopic ultrasonography (EUS), computed tomography (CT) of the thorax and abdomen with contrast and ultrasonography of the cervical region with fine-needle aspiration cytology for any suspicious nodes. Positron emission tomography-computed tomography (PET-CT) will be used when the disease stage is difficult to confirm by general imaging examination, but it is not compulsory.

### Interventions

#### Standard oesophagectomy

Standard oesophagectomy surgery will be performed for patients by specialists. The surgical approach to the mid or lower thoracic oesophagus will be standardised to two-stage oesophagectomy to achieve a 5-cm minimum proximal margin. For tumours located over the proximal mid thoracic oesophagus where a 5-cm proximal margin cannot be achieved, a three-stage oesophagectomy will be performed. We will perform a two-field lymphadenectomy in situations of either cervical or thoracic anastomosis. All the oesophagectomies will be performed through the thoracoscopy operation or an open approach. A radical surgical resection is defined as macroscopic clearance of the oesophageal tumour with no residual disease left (R0). Patients in the standard oesophagectomy group will receive postoperative adjuvant chemotherapy if the resection is considered to be R1, i.e. microscopic disease is left behind.

#### Chemoradiotherapy

##### Radiotherapy

Intensity-modulated radiation therapy (IMRT) will be performed for patients in the DCRT group with 50 Gy/25 F and in the surgery group with 42 Gy/21 F for NCRT, at 2 Gy/day, five times/week, until disease progression or unacceptable toxicity is found. The dosage for the individual patients will be governed by the dose constraints of the normal organs. The target volume length includes 5 cm on each side of the imaged visible tumour and malignant nodes. Radiotherapy will be delivered in two consecutive phases. Phase I starts with anterior-posterior opposing portals to 30 Gy, while phase II will be given with three fields to another 20 Gy (or 12 Gy in the surgery group for NCRT), which is subject to the limiting radiation dose of the heart, lung and spinal cord.

##### Chemotherapy

Patients will be randomised to one of following three regimens:

1. Xelox: oxaliplatin 65 mg/m^2^, d1, 8, 22, 29, plus capecitabine, 625 mg/m^2^, bid, d1–5; 6 weeks in total
2. Single capecitabine: capecitabine, 625 mg/m^2^, bid, d1–5; q1w, 6 weeks in total
3. PF: cisplatin, 75 mg/m^2^, d1, 29, 5-FU, 750 mg/m^2^, CIV 24 h, d1–4, d29–32.

#### Outcome measures

The primary outcome measures are 2-year/5-year overall survival (OS). The secondary outcomes are 2-year/5-year disease-free survival (DFS), treatment-related AEs and QoL. The recurrence of the disease is defined as either endoscopic recurrence confirmed with biopsy or distant metastasis. The operative mortality is defined as an in-hospital death within 30 days of the perioperative period.

#### Follow-up

All patients will be followed in the hospital where they received treatment at the 16th week after random allocation and then at 3-month intervals in the first 2 years and at 6-month intervals for the next 3 years thereafter. Local or systemic recurrences and any AEs will be recorded. For the DCRT group, patients can be treated with salvage oesophagectomy if the disease does not reach complete remission at the 16th week follow-up. For the surgery group as well, patients can be treated with radiation or chemotherapy if R0 is impossible to reach or there is local recurrence at the 16th week of follow-up.

**Fig. 2:**
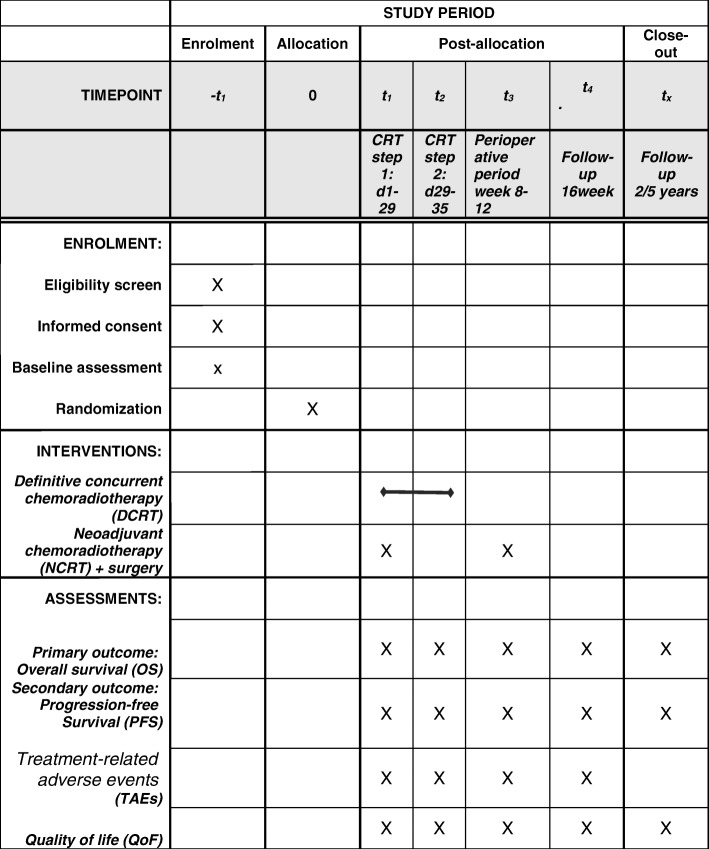
Schedule of enrolment, interventions and assessments

QoL will be evaluated in all patients using the Quality of Life Questionnaire-Core 30 (QLQ-C30 version 3.0, in Chinese) and the supplemental Quality of Life-Oesophageal Module 18 Questionnaire (QLQ-ES18, in Chinese) for patients with EC, both of which were developed by the European Organisation for Research and Treatment of Cancer (EORTC). For this evaluation, each patient will be visited in person during hospitalisation 1 week before and 1 week after surgery and contacted by telephone at 12 and 24 weeks postoperatively (Fig. 2) [17].

### Data management

This trial will be conducted in accordance with International Conference on Harmonisation (ICH) Guidelines for Good Clinical Research Practice and relevant local ethical regulations. Study data will be collected and managed using a regulatory approved electronic data capture system.

Data quality will be assured through range checks for data values. Integrity of trial data will be monitored by regularly scrutinising data for omissions and errors. In order to protect confidentiality before, during and after the trial, personal information about potential and enrolled participants will remain secure in a locked research office at the First Affiliated Hospital of HUST. Study data will be retained, securely password protected, for a minimum of 15 years from completion. Details of data management procedures can be found in the protocol.

### Data analyses

The primary method of analysis will be provision of descriptive statistics characterising key features, such as recruitment rate (number of patients approached, number consenting to participate and number eligible to be randomised), as well as frequencies and proportions of missing data and participant attrition, during both intervention and follow-up periods every 3 months.

### Sample size estimation and statistical analysis

Sample size calculation is based on the 5-year OS rates: 29.4% in patients treated with oesophagectomy and 50% in the DCRT group [18]. We used the log-rank test to compare the survival rate difference between the surgery and DCRT group. We defined α as 0.05 and β as 0.2. We supposed that the rate of loss to follow-up per year is 2.5% and 12.5% in 5 years. A sample size of 96 patients was determined to be required for each group.

The primary and secondary outcome measures will be compared using Student’s *t* test for the normally distributed data and the Mann-Whitney *U* test for the nonparametric data. For the data in proportions, a chi-squared test or Fisher’s exact test (if one of the expected values is less than 5) will be used. The provision of a 95% confidence interval will be calculated with the relative risk for cancer recurrence, morbidities and mortalities related to each therapy. We will use the Kaplan-Meier curve to represent the probability of survival within 2 years and 5 years after the initial diagnosis, and compare the two groups using the log-rank test. A value of *p* < 0.05 is considered to be statistically significant. The statistical analysis will be performed with the SPSS software (version 13.0; SPSS Inc., Chicago, IL, USA).

### Monitoring

#### Collecting, assessing, reporting and managing adverse events

The most common side effects of CRT are myelosuppression, oral mucositis, hand-foot syndrome and peripheral neuritis. More severe side effects are rare. Information about solicited and spontaneously reported AEs will be sought from all participants during telephone reviews by the trial General Practitioner/General Investigator (GP). If a participant reports an AE, the trial GP will determine appropriate action, which may include dose alteration or withdrawal. If an AE is identified as more serious than grade 4, the trial GP will forward this information immediately to the Principal Investigator and Data Safety Monitoring Board. All of the serious AEs (SAEs), suspected adverse reactions and serious suspected unexpected adverse reactions will be recorded immediately in the source documents and on the AE case report form. Each event will be followed until resolution or stabilisation or until it has been determined that the study treatment is not causal. SAEs still ongoing at the end of the study will be followed up to determine final outcome. Any SAE that occurs after the study will be recorded and reported immediately and considered to be possibly related to the study treatment. Economic compensation will be provided by the trial sponsor to those who suffer harm from the trial participation.

For the data monitoring of the QoL outcome, firstly, some measures will be taken to prevent and reduce missing data by enhancing investigator training, communication, patient education and data monitoring. Secondly, we will confirm the causes of missing data case by case and record them in detail. Finally, suitable missing data handling methods such as last observation carried forward (LOCF) or multiple imputation (MI) will be performed.

#### Dissemination

Authorship eligibility guidelines will follow International Committee of Medical Journal Editors (ICMJE) guidelines. The final trial dataset will be available to the investigative team and on reasonable request.

## Discussion

This is the first registered prospective head-to-head clinical trial to compare the outcomes between radical operation and DCRT in patients with ESCC in the highest incidence area worldwide. In the current international guidelines for EC, DCRT is recommended as an effective intervention approach only for patents with local advanced disease but who are not suitable for oesophagectomy. The reasons could be the patient’s willingness, poor performance status, concomitant cardiopulmonary disease and so on. Some studies reported that patients received a survival benefit from DCRT. However, prospective clinical trials that compare DCRT and NCRT plus surgery through a head-to-head method are still limited. Also, the participants who were reported in published studies were mainly western Caucasian patients with oesophageal adenocarcinoma. Sjoquist et al. reported that the EC patients with adenocarcinoma pathology had a higher disease regression rate than those with squamous cell cancer after NCRT [18]. The long-term survival status of patients with ESCC after DCRT or NCRT plus surgery treatment is unclear.

Researchers from Hong Kong University initiated an excellent prospective clinical trial to compare the long-term outcomes between DCRT and surgery for ESCC patients. In this study, the overall 5-year survival favours CRT, but the difference did not reach statistical significance (surgery 29.4% and CRT 50%, *p* = 0.147) [13, 14]. The intervention of chemoradiotherapy used in this study is cisplatin 60 mg/m^2^ with hydration therapy given on days 1 and 22, whereas 5-FU is administered as a continuous infusion at 200 mg/m^2^/day from day 1 to day 42. Radiotherapy was delivered as three-dimensional conformal radiation therapy (3DCRT) with a total of 50–60 Gy given in 25–30 fractions over 5–6 weeks. It is inconvenient for patients to be administered 5-FU continuously for 42 days, while IMRT has been reported as more effective and tolerable than 3DCRT. Therefore, we designed this study to investigate the role of DCRT compared with NCRT followed by radical operation in patients with locally advanced ESCC in the highest incidence area worldwide using IMRT (50 Gy/25 F) and different chemotherapy regimens (capecitabine, Xelox, PF, randomised delivery). In the pilot trial, 86 patients finished 16 weeks of follow-up with at least these three regimens in the DCRT group (capecitabine:Xelox:PF = 24:37:25) [19, 20]. The incidences of grade 3–5 AE were 25%, 32.4% and 64% (*p* = 0.03) and the pathological complete response (pCR) rates were 50%, 48.6% and 48% in the three subgroups, respectively (*p* = 0.99). Additionally, objective response rates (ORRs) of 87.5% (21/24), 83.8% (31/37) and 100% (25/25), respectively (*p* = 0.133) were observed. No differences were seen in the complete response (CR) and ORR between the three subgroups. Therefore, it is worth exploring the roles of both the DCRT and single capecitabine in CRT in patients with advanced ESCC using a larger sample size.

### Trial status

The trial began recruitment in April 2017. Participants will be recruited until December 2020, if necessary.

## Additional file

Additional file 1: SPIRIT 2013 checklist. (DOC 133 kb)

## Abbreviations

3DCRT: Three-dimensional conformal radiation therapy
5-FU: 5-Fluorouracil
AE: Adverse event
CRT: Concurrent chemoradiotherapy
CT: Computed tomography
DCRT: Definitive CRT
EC: Oesophageal cancer
EORTC: European Organisation for Research and Treatment of Cancer
ESCC: Oesophageal squamous cell carcinoma
EUS: Endoscopic ultrasonography
F: Fraction
IMRT: Intensity-modulated radiation therapy
NCRT: Neoadjuvant CRT
OS: Overall survival
PFS: Progression-free survival
QLQ-C30: Quality of Life Questionnaire-Core 30
QLQ-ES18: Quality of Life-Oesophageal Module 18 Questionnaire
QoL: Quality of life
